# MRI detection of suspected nasopharyngeal carcinoma: a systematic review and meta-analysis

**DOI:** 10.1007/s00234-022-02941-w

**Published:** 2022-04-30

**Authors:** Vineet Vijay Gorolay, Naomi Natasha Niles, Ya Ruth Huo, Navid Ahmadi, Kate Hanneman, Elizabeth Thompson, Michael Vinchill Chan

**Affiliations:** 1 Department of Radiology, Royal Price Alfred Hospital, University of Sydney, Sydney, NSW Australia; 2 Department of Ear, Nose and Throat Surgery, Concord Hospital, Concord, NSW Australia; 3 Department of Radiology, Hospital Road, Concord Repatriation and General Hospital, University of Sydney, Concord, NSW 2139 Australia; 4 Department of Ear, Nose and Throat Surgery, Royal Prince Alfred Hospital, University of New South Wales, Sydney, NSW Australia; 5 Department of Medical Imaging, Peter Munk Cardiac Center, University Health Network, University of Toronto, Toronto, ON Canada

**Keywords:** MRI, Nasopharyngeal carcinoma, Biopsy

## Abstract

**Purpose:**

Endoscopic biopsy is recommended for diagnosis of nasopharyngeal carcinoma (NPC). A proportion of lesions are hidden from endoscopic view but detected with magnetic resonance imaging (MRI). This systematic review and meta-analysis investigated the diagnostic performance of MRI for detection of NPC.

**Methods:**

An electronic search of twelve databases and registries was performed. Studies were included if they compared the diagnostic accuracy of MRI to a reference standard (histopathology) in patients suspected of having NPC. The primary outcome was accuracy for detection of NPC. Random-effects models were used to pool outcomes for sensitivity, specificity, and positive and negative likelihood ratio (LR). Bias and applicability were assessed using the modified QUADAS-2 tool.

**Results:**

Nine studies were included involving 1736 patients of whom 337 were diagnosed with NPC. MRI demonstrated a pooled sensitivity of 98.1% (95% *CI* 95.2–99.3%), specificity of 91.7% (95% *CI* 88.3–94.2%), negative LR of 0.02 (95% *CI* 0.01–0.05), and positive LR of 11.9 (95% *CI* 8.35–16.81) for detection of NPC. Most studies were performed in regions where NPC is endemic, and there was a risk of selection bias due to inclusion of retrospective studies and one case–control study. There was limited reporting of study randomization strategy.

**Conclusion:**

This study demonstrates that MRI has a high pooled sensitivity, specificity, and negative predictive value for detection of NPC. MRI may be useful for lesion detection prior to endoscopic biopsy and aid the decision to avoid biopsy in patients with a low post-test probability of disease.

## Introduction

### Rationale

Nasopharyngeal carcinoma (NPC) has unique epidemiology, natural history, and treatment response necessitating distinct management paradigms [1]. It encompasses keratinizing, non-keratinizing, and basaloid type squamous cell carcinomas [2]. The non-keratinizing subtype is most common in endemic populations, including east and southeast Asia, north Africa, and the Arctic [3]. The global incidence of NPC has been increasing between 2009 and 2019, although mortality and morbidity has reduced over this period [4]. Major risk factors include genetic and family history, environmental agents such as nitrosamines in preserved foods, and exposure to formaldehyde, wood dusts, and fumes [5]. Epstein-Barr virus (EBV) infection is closely associated with NPC but its exact role in pathogenesis remains enigmatic [3, 5].

Biopsy of the primary tumor is required for definitive diagnosis [6] and is generally performed endoscopically under local or general anesthesia [7]. Although endoscopic biopsy is the recommended first step across guidelines [1, 6–8], some 10% of nasopharyngeal cancers are missed at initial endoscopy. This is generally attributed to small size, submucosal location [9], coexistent hyperplasia [10], and anatomic difficulty in assessing the lateral pharyngeal recess [11]. For endoscopically occult cases, current guidelines recommend repeat endoscopy and biopsy of tissue identified as abnormal on MRI or PET-CT [6].

MRI is established as the preferred modality for locoregional staging of NPC [3, 6, 7, 10, 12], due to good soft tissue visualization of parapharyngeal or masticator space involvement, perineural and intracranial spread [8, 13] with PET-CT playing a complementary role in staging of nodal and distant metastasis [14, 15]. MRI has not historically been used for primary diagnosis due to concerns about poor sensitivity for small mucosal lesions [16].

Unfortunately, the vast majority of patients present with advanced disease, with less than 8% presenting at stage I [17]. Beyond stage II, there is a substantial rate of distant metastasis [8], for which concurrent chemoradiotherapy is often indicated [3, 6, 7]. The addition of chemotherapy increases risk of acute toxicity [3]. Consequently, there has been substantial interest in screening for early NPC with EBV immunoserology, in some studies coupled with MRI [3, 18]. This has renewed interest in the role and diagnostic accuracy of MRI, with recent efforts to produce MRI diagnostic criteria [19, 20].

### Objectives

The primary endpoint of this systematic review and meta-analysis is to pool the sensitivity, specificity, likelihood ratios (LRs), and hierarchical summary receiver-operating characteristics (HSROC) of MRI for primary detection of nasopharyngeal carcinoma relative to the reference standard of post-MRI endoscopic biopsy. Secondary endpoints are to identify barriers by way of qualitative or quantitative factors that influence diagnostic accuracy.

## Materials and methods

### Search strategy

The search strategy was devised in accordance with the revised PRISMA 2020 statement [21] and registered with PROSPERO (CRD42021252609) [22]. Twelve databases and registries were searched electronically including Ovid MEDLINE, Embase, Cochrane Database of Systematic Reviews, Cochrane Central Register of Controlled Trials, Cochrane Clinical Answers, Cochrane Methodology Register, American College of Physicians Journal Club, Database of Abstracts of Reviews of Effects, NHS Economic Evaluation Database, PubMed, PROSPERO, and Google Scholar. Search terms included (“magnetic resonance imaging (MRI)”) and (“nasopharyngeal carcinoma,” “nasopharyngeal cancer,” “nasopharynx cancer,” and “nasopharyngeal neoplasm”) as well as relevant truncations, MeSH terms, and keywords. Articles published between inception and March 2022 were included, without language restrictions. Duplicate studies were removed, and titles and abstracts were screened independently by 2 reviewers (VVG and NNN). Full texts of potentially relevant studies were obtained, and their reference lists reviewed for any additional studies.

### Selection criteria

**Fig. 1:**
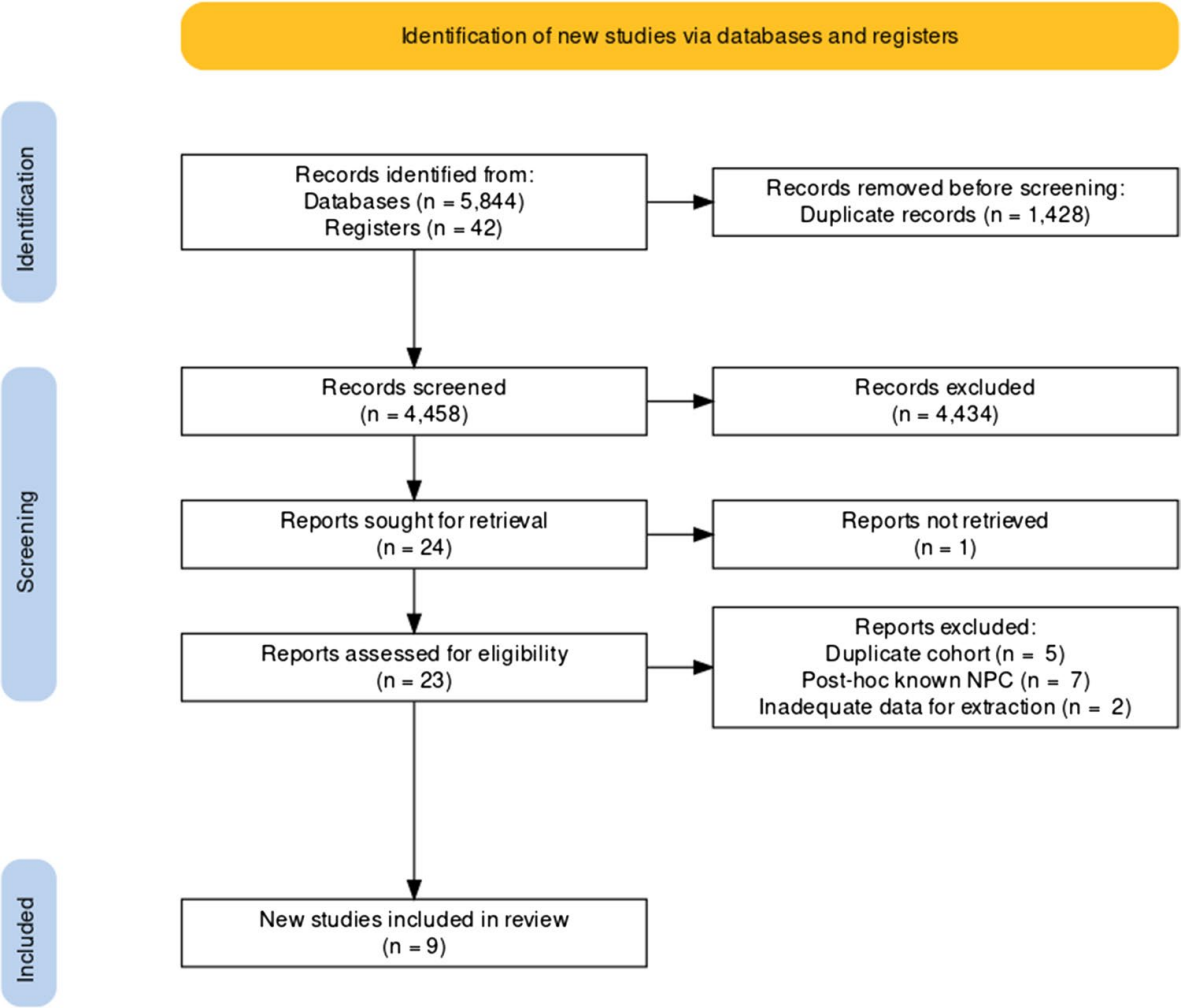
PRISMA flow diagram for study selection [21, 23]

We included prospective and retrospective studies that assessed performance of MRI for diagnosis of NPC in patients with suspected disease, compared to histopathology as the reference standard. Following full-text review, articles were included if they reported sensitivity and specificity values for detection of primary NPC. Papers for which this was not possible were excluded. Articles which assessed accuracy for detection of recurrent tumor were excluded. Studies with risk of overlapping cohorts were scrutinized and the most complete dataset was included to avoid duplication of data. Abstracts, case reports, case series, editorials, and prior systematic reviews or meta-analyses were also excluded due to risk of publication bias and duplication. The selection process was summarized graphically per the PRISMA [21] guidelines using the ShinyApp tool [23] in Fig. 1.

### Data extraction

Two authors (VVG and NNN) independently extracted data including study design, patient demographics, inclusion and exclusion criteria, cohort enrolment and MRI-technique (field strength, sequences), diagnostic criteria utilized, and diagnostic accuracy data (true- and false-positives and negatives, sensitivity, specificity, and positive and negative predictive values). If multiple diagnostic features were assessed, we included the parameter with best diagnostic performance in the meta-analysis. If data were ambiguous or could not be extracted into 2 × 2 tables, the authors of the paper in question were contacted for clarification where possible. Discrepancies between the 2 reviewers were resolved by discussion and consensus, and results reviewed by the senior investigator (MVC).

### Methodologic quality assessment

Included studies were assessed using the revised Quality Assessment of Diagnostic Accuracy of Studies (QUADAS-2) tool [24]. This tool facilitates critical assessment of risk of bias and applicability concerns for the index and reference test for each included study, as well as methodologic assessment of study design and patient selection techniques. For each assessment, the level of risk is designated as low, high, or unclear.

### Statistical analysis

Statistical analysis was performed using STATA v14.1 (StataCorp, College Station, TX). A two-tailed *p*-value < 0.05 was considered statistically significant. To pool diagnostic accuracy measures, we used a bivariate mixed-effects regression model that allows correlation between sensitivity and specificity. Results were presented as summary sensitivities, specificities, LRs, and ROC AUC values with 95% CIs. A positive *LR* > 10 or a negative *LR* < 0.1 was considered to be strong diagnostic evidence [25].

Interstudy variability was assumed, and an exploration of the causes of variability, including study design (prospective vs. retrospective) differences, was performed using meta-regression. Statistical analyses of variability and publication bias are not included in this report in accordance with updated recommendations by the PRISMA Diagnostic Test Accuracy Group [26]. The area under the HSROC curve was calculated, using a ≥ 0.9 threshold to indicate high test performance accuracy [27].

## Results

### Literature search

A total of 5886 references were identified through searches of 12 electronic databases and registers, of which 23 met criteria for full-text review. Manual search through reference lists yielded 1 additional relevant study. Following exclusion of studies with overlapping cohorts (*n* = 5), post hoc assessment of known nasopharyngeal tumor (*n* = 7), or for whom data could not be extracted into 2 × 2 tables (*n* = 2) or a full text could not be obtained (*n* = 1), there were 9 eligible studies for the meta-analysis. A summary of the study selection process according to the PRISMA format is presented in Fig. 1.

### Study characteristics

All 9 included studies were single-institution studies, of which 4 were prospective [28–31] and 5 were retrospective [16, 32–34]. A total of 1736 patients with suspected disease underwent MRI and had histopathologic assessment, of whom 337 had a confirmed diagnosis of nasopharyngeal carcinoma. Approximately 38% of patients were female, with age ranging from 10 to 86 years.

All studies included patients suspected of having nasopharyngeal carcinoma, due to positive EBV serology, middle ear effusion, blood-stained epistaxis or rhinorrhea, cervical nodal metastases, or other similar clinical features [16, 30, 32, 33, 35]. One study included patients who presented with cervical nodal metastasis from a presumed head and neck primary source which was occult on initial clinical examination and endoscopy [34]. One study recruited from a high-risk screening cohort with positive EBV serology [28]. One case–control study included patients with proven or suspected NPC [16]; only the cohort suspected to have NPC at the time of MRI were included in our pooled analysis. All studies excluded patients for whom both imaging and histopathology were not obtained. Four studies [28–30, 33] excluded non-NPC primary tumors from analysis. Two studies [28, 34] excluded patients with a history of other malignancy. One study [33] excluded NPC beyond radiologic stage T1.

Two studies did not report MRI field strength or technique [32, 35]. MRI was performed at field strength of 3 T in three studies [28, 33, 34], 1.5 T in three studies [16, 29, 30], and either 1.0 or 1.5 T in one study [31]. All studies where MRI technique was listed included T1-weighted pre- and post-gadolinium spin-echo, and T2-weighted sequences with or without fat suppression. Three studies [28, 31, 34] included post-gadolinium volumetric gradient echo T1-weighted sequences. Slice thickness varied between 1 and 5 mm with intersection gaps of either 0 or 1 mm [16, 28–31, 33, 34] with two studies not reporting these variables [32, 35]. Three studies [16, 28, 30] used a multiparametric scoring system to assess likelihood of NPC, one study [33] assessed diagnostic performance of discrete imaging findings (e.g., nasopharyngeal asymmetry) and the remainder did not specify MRI diagnostic criteria.

Histopathology was used as the reference standard for NPC diagnosis in all studies. In cases where initial endoscopy was negative, repeat biopsy targeted to the imaging abnormality was used for definitive diagnosis in four studies [16, 28–30]. The remaining studies did not report whether biopsy was blinded to MRI results. One study [31] included surgical resection specimens in addition to endoscopic biopsy. One study included follow-up histopathology verification from a local cancer registry [28]. Absence of NPC was diagnosed by benign findings at biopsy in seven studies [29–35]. In the two remaining studies [16, 28], NPC was considered absent if initial endoscopy and MRI were both normal and no new abnormality was detected at clinical and imaging follow-up.

A complete summary of study details, MRI technique and patient baseline characteristics are presented in Table 1.

**Table 1:** Study and cohort characteristics

Author year	Location	Cohort	Study period	Patients (*n*)	NPC	Female (%)	Age mean (range)	EBV + ve	Symptomatic	MRI vendor (T)	T1 NC	T1 + C	T2 SE	T2FS or STIR	Thick, gap (mm)	MRI criteria	Reference standard
Liu 2021	Hong Kong	Prospective	2014–2018	720	24	56	53 (NR)	720	NR	Siemens^a^ (3.0)	Y	Y	Y	N	3, 1	Grading	Endoscopic biopsy or clinical follow-up
Shayah 2019	United Kingdom	Retrospective	2009–2017	42	10	27	52 (17–86)	NR	NR	NR	NR	NR	NR	NR	NR, NR	NR	Endoscopic biopsy
Yoo 2018	Korea	Retrospective	2011–2016	73	7	16	57 (33–79)	NR	73	Philips^b,c^ (3.0)	Y	Y	N	Y	1–3, 0	NR	Endoscopic biopsy
Wang 2017	China	Retrospective	2013–2016	124	38	49	51 (NR)	NR	120	Philips^d^, Siemens^a^ (3.0)	Y	Y	Y	N	2–5, NR	NP asymmetry	Endoscopic biopsy
Bercin 2017	Turkey	Retrospective	2010–2014	199	14	39	39 (10–85)	NR	NR	NR	NR	NR	NR	NR	NR, NR	NR	Endoscopic biopsy
Gao 2014	China	Prospective	2010–2012	150	71	34	48 (21–68)	NR	NR	GE (1.5)	Y	Y	Y	N	4, 1	NR	Endoscopic biopsy
King 2011	Hong Kong	Prospective	2007–2010	246	77	40	50 (17–85)	122	205	Philips^b,e^, Siemens^a^ (1.5)	Y	Y	N	Y	4, 0	Grading	Endoscopic biopsy
King 2006	Hong Kong	Retrospective	1996–2005	77	3	61	47 (18–84)	17	60	Philips^e^ (1.5)	Y	Y	Y	Y	4, 0	Grading	Endoscopic biopsy or MRI follow-up
Held 1994	Germany	Prospective	NR	105	93	NR	NR	NR	NR	Siemens^a^ (1.0, 1.5)	Y	Y	Y	N	3–5, NR	NR	Histopathology

MRI Vendors: ^a^Siemens Magnetom; ^b^Philips Intera Achieva; ^c^Philips Achieva TX; ^d^Philips Ingenia; ^e^Philips Gyroscan. Abbreviations: *EBV* + *ve*, positive EBV serology; *T1NC*, T1-weighted non-contrast; *T1* + *C*, T1-weighted post-contrast; *T2FS*, T2-weighted fat-suppressed; *STIR*, short tau inversion recovery; Thick, slice thickness; Gap, intersection gap; *NP*, nasopharynx; *NR*, not reported

### Methodologic quality

Potential study bias was assessed using the QUADAS-2 metric [24, 36]. Three studies were deemed at risk of selection bias due to case–control design [33], limited description of inclusion [31] or exclusion [35] criteria. Seven studies [16, 29–33, 35] did not clearly describe whether patients were recruited in a randomized or consecutive manner, creating an unclear risk of selection bias. In general, the spectrum of patients was representative of patients who would be investigated with endoscopic biopsy in practice. In most studies, MRI technique and interpretation criteria were well described, with interpretation blinded to endoscopic findings. Two studies [32, 35] did not describe MRI diagnostic criteria. In two studies [16, 28], patients with no lesion detected at MRI underwent follow-up imaging and clinical assessment instead of biopsy. In two studies [28, 34], endoscopy and histopathology interpretation were not blinded to MRI findings. Two studies [29, 32] did not report indeterminate or uninterpretable results and three studies [31, 32, 35] did not discuss patient withdrawals. Only one study [28] specified the time interval between MRI and endoscopic biopsy. A complete summary of the QUADAS-2 metrics is included in Table 2. There was uncertainty about applicability of selected cohort in four studies [31, 33–35] and regarding applicability of the index test in two studies [32, 35]. Concerns regarding applicability are summarized in Table 3.

**Table 2:** QUADAS-2 assessment of study bias [24]

Author	Patient selection					Index				Reference			Flow & timing			
Year	1	2	3	4	5	6	7	8	9	10	11	12	13	14	15	16
Liu 2021	Y	Y	Y	Y	Y	Y	Y	N	Y	Y	Y	U	Y	N	Y	Y
Shayah 2019	U	Y	Y	Y	Y	U	N	Y	Y	Y	Y	U	U	Y	N	N
Yoo 2018	Y	Y	Y	Y	Y	Y	Y	Y	Y	Y	Y	N	U	Y	Y	Y
Wang 2017	U	N	Y	Y	Y	Y	Y	Y	Y	Y	Y	U	U	Y	Y	Y
Bercin 2017	U	Y	Y	Y	N	U	N	Y	U	Y	Y	Y	U	Y	Y	N
Gao 2014	U	Y	Y	Y	Y	Y	U	Y	Y	Y	Y	Y	U	Y	N	Y
King 2011	U	Y	Y	Y	Y	Y	Y	Y	Y	Y	Y	Y	U	Y	Y	Y
King 2006	U	Y	Y	Y	Y	Y	Y	N	Y	Y	Y	Y	U	N	Y	Y
Held 1994	U	Y	N	Y	Y	Y	Y	Y	U	Y	Y	Y	U	Y	Y	N

Domain 1: patient selection. (1) Was a consecutive or random sample of patients enrolled? (2) Was a case–control design avoided? (3) Were patient selection criteria clearly described? (4) Were the patients selected appropriate for the tests in clinical practice? (5) Were patient exclusion criteria clearly described? Domain 2: index test. (6) Was the index test performed in a clearly described and reproducible manner? (7) Were criteria for interpretation of the index test clear and reproducible? (8) Did all patients investigated with the index test also have confirmation with the reference test? (9) Were the index test results interpreted without knowledge of the reference standard results? Domain 3: reference test. (10) Is the reference standard appropriate for correctly classifying the condition? (11) Was the reference standard performed in a clearly described and reproducible manner? (12) Were the reference standard results interpreted without the knowledge of the index test results? Domain 4: flow and timing. (13) Was there an appropriate time interval between the index test and the reference standard? (14) Were all patients investigated with the reference test? (15) Were intermediate and uninterpretable results reported? (16) Were patient withdrawals discussed? Abbreviations: *Y *yes, *N *no, *U *unclear

**Table 3:** Assessment of concerns regarding applicability [24]

Author	Applicability		
Year	Selection	Index	Reference
Liu 2021	N	N	N
Shayah 2019	N	Y	N
Yoo 2018	U	N	N
Wang 2017	U	N	N
Bercin 2017	U	N	N
Gao 2014	N	N	N
King 2011	N	N	N
King 2006	N	N	N
Held 1994	U	N	N

Domain 1: patient selection. Are there concerns that the included patients do not match the review question? Domain 2: index test. Are there concerns that the index test, its conduct, or interpretation differ from the review question? Domain 3: reference test. Are there concerns that the target condition as defined by the reference standard does not match the review question? Abbreviations: *Y* yes, *N* no, *U* unclear

### Diagnostic accuracy of MRI

**Fig. 2:**
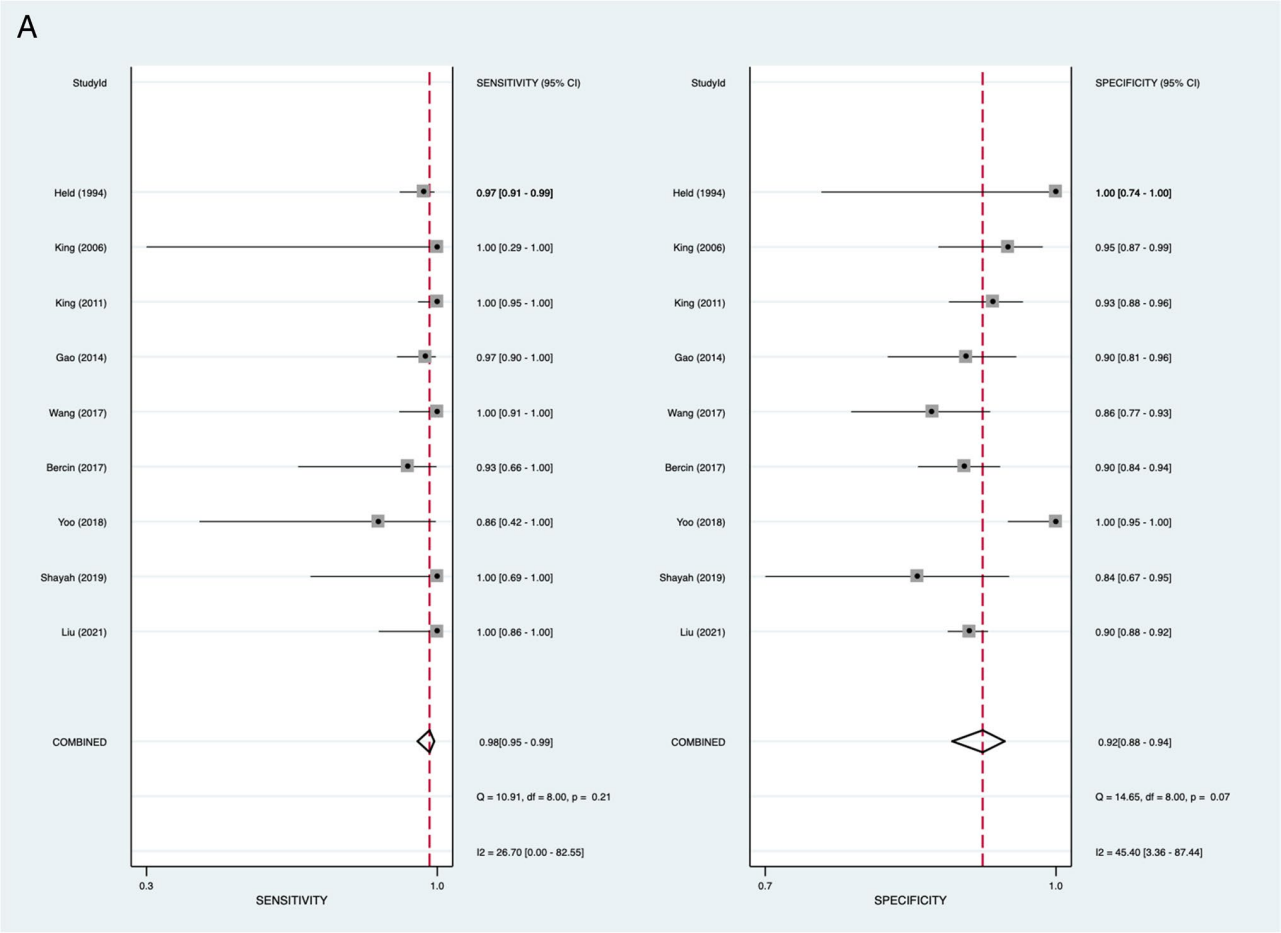

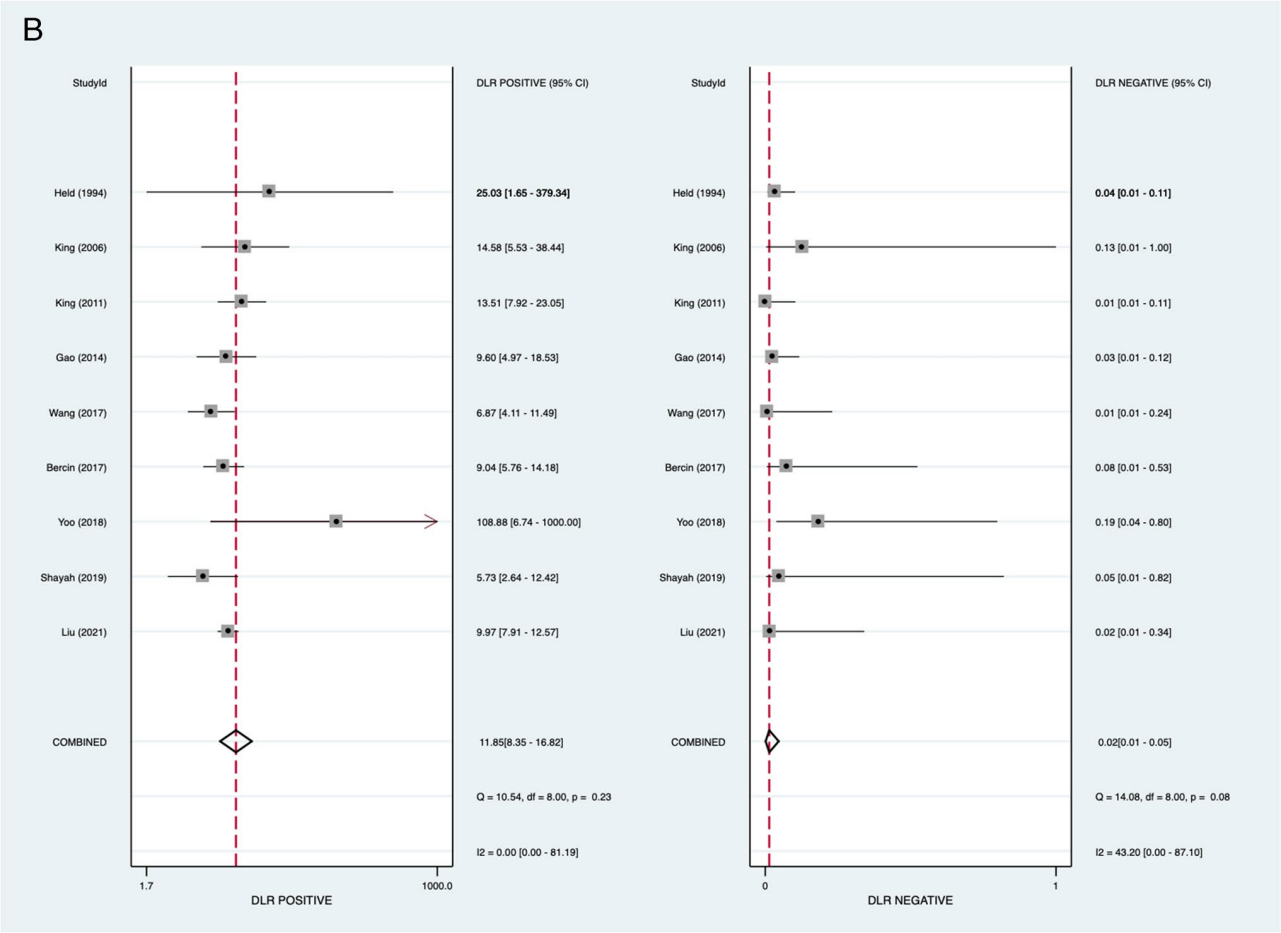

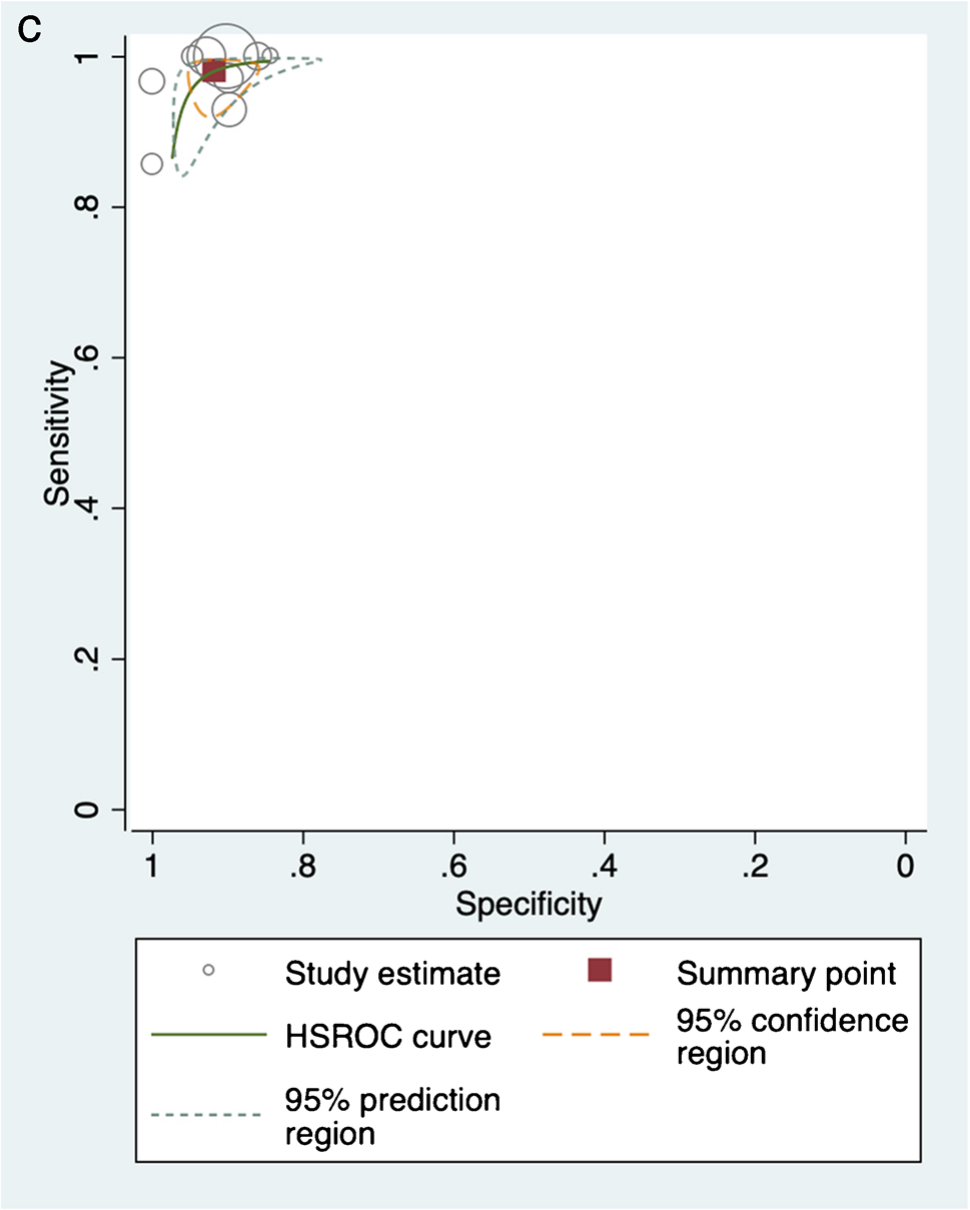
Pooled analysis of included studies. **A** Pooled sensitivity and specificity. **B** Pooled positive and negative likelihood ratios. **C** Hierarchical summary receiver operator curve (HSROC)

MRI assessment for nasopharyngeal carcinoma demonstrated a pooled sensitivity of 98.1% (95% *CI* 95.2–99.3%), specificity of 91.7% (95% *CI* 88.3–94.2%), negative LR of 0.02 (95% *CI* 0.01–0.05), and positive LR of 11.9 (95% *CI* 8.35–16.81). The area under the curve (AUC) of the sROC was 0.98 (95% *CI* 0.97–0.99) (Fig. 2).

### Subgroup analysis

Study design (retrospective vs. prospective) did not demonstrate a significant impact on diagnostic sensitivity (*p* = 0.48) or specificity (*p* = 0.83) in meta-regression models. There was insufficient data regarding lesion size or tumor stage to facilitate further subgroup analysis.

### Other qualitative or quantitative findings

Although MRI was generally performed prior to biopsy, only one study [28] reported the time elapsed between MRI and endoscopic biopsy (median 1.8 months). The most reported cause of false positive MRI was nasopharyngeal hyperplasia within the pharyngeal recess [16, 29, 37]. False negatives were attributed to motion degraded sequences [31] or lesions < 10 mm in size [34].

## Discussion

Nasendoscopy and biopsy have long been established as the gold standard for diagnosing NPC. However, NPC may be submucosal or contained within the pharyngeal recess and thus difficult to identify at endoscopy [11], resulting in delayed diagnosis and treatment [16]. Recent meta-analyses [14, 15] have supported current practice guidelines in which MRI is employed for locoregional staging, with PET-CT playing a key role in staging of nodal and distant metastases [6, 8, 12, 38]. However, the role of MRI in primary diagnosis of NPC is yet to be established.

Our meta-analysis supports the use of MRI as an accurate primary diagnostic tool, with a pooled sensitivity of 98.1%. MRI was also found to have excellent specificity and negative LR. MRI-detected abnormalities could direct biopsy planning whereas a normal MRI examination would establish a low post-test probability of disease. The applicability of these findings is strengthened by the MRI protocols involved, which included T2-weighted and pre- and post-gadolinium T1-weighted sequences on 1, 1.5 and 3 T magnets at slice thicknesses between 1 and 5 mm, reflecting real-world heterogeneity of protocols in clinical practice [13].

The implication for current guidelines is that MRI could be the initial diagnostic test prior to endoscopic biopsy. Pre-operative MRI may increase the yield of endoscopic biopsy by alerting the surgeon to multiple lesions or lesions which may be endoscopically occult, and aid selection of patients who may benefit from intraoperative image-guided stereotactic approaches. The negative LR of MRI implies greatly reduced post-test probability of disease. MRI is a non-invasive technique which does not require anesthesia, yields excellent soft-tissue characterization of the lateral pharyngeal recess, submucosal soft tissues, and retropharyngeal nodes [13], and already has an established role in locoregional staging [6, 14, 15]. This raises the potential for use of MRI to exclude disease, reducing need for invasive biopsy.

The major pitfall for diagnosis highlighted in the included studies is false-positive results due to asymmetric nasopharyngeal hyperplasia [39], reported in up to 14% of cases [34]. Authors have attempted to distinguish this condition from NPC by assessing features such as lesion subsite, size, signal characteristics [33], and anatomic features such as the deep mucosal white line [19, 33]. Advanced imaging techniques [40] have also been investigated as potential discriminators [41–43]. Notably, several papers by King and colleagues [19, 20], some of which are included in this meta-analysis [16, 28, 30], have developed and applied multiparametric diagnostic criteria. Conversely, several included papers did not describe criteria for a positive test [29, 31, 32, 34, 35], limiting potential for more subgroup analysis. Lesion size is known to limit endoscopic detection sensitivity [9]. Unfortunately, there was insufficient data among the included papers to allow subgroup analysis of MRI accuracy based on lesion size or tumor stage. Further comparative studies are required for prospective validation of such grading systems.

The results of this systematic review and meta-analysis should be considered in context of several limitations. Only two studies [28, 34] reported whether patients were enrolled randomly or consecutively, with one case–control study [33] included. Combined with the inclusion of retrospective studies, and of multiple studies from a single institution, this introduces risk of selection and publication bias. Two studies [16, 28] used clinical and imaging follow-up to document absence of NPC. Although such differential verification may overestimate test accuracy, it is difficult to ethically justify biopsy in patients with no visible lesion [44]. One study [34] was deemed at high risk of diagnostic review bias [44] because endoscopy or histopathology interpretation was not blinded to MRI findings. When the initial endoscopy is negative, repeat endoscopy and biopsy targeted to imaging abnormalities is already the established standard of care [6]. Furthermore, in clinical practice, histopathologic verification of suspected malignancy is still necessary and often interpreted with awareness of prior test results, such that the requirement for blinding is not so justifiable [44]. Only one study reported time elapsed between MRI and biopsy [28], whereas the others introduce risk of ascertainment or disease progression bias [44]. Finally, the included cohort from the available literature is relatively small. Further prospective studies could assess the most robust sequences and imaging characteristics to enhance diagnostic accuracy.

## Conclusion

Gadolinium-enhanced nasopharynx MRI demonstrates exceptional sensitivity, positive and negative LR for diagnosis of nasopharyngeal carcinoma with good specificity. The results support use of MRI as a primary diagnostic tool for lesion detection, in addition to its established role in locoregional staging. If used prior to endoscopic biopsy, it may increase the yield of biopsy or aid the decision to avoid biopsy in patients with low post-test probability of disease.

## Data Availability

Data and materials are sourced from published medical journals which have been referenced in the manuscript.

## Abbreviations

MRI: Magnetic resonance imaging
NPC: Nasopharyngeal carcinoma
CI: Confidence interval
LR: Likelihood ratio
EBV: Epstein-Barr virus
HSROC: Hierarchical summary receiver-operating characteristics
QUADAS-2: Quality Assessment of Diagnostic Accuracy of Studies Version 2

## Code availability

Custom code in SAS.

All authors certify that they have no affiliations with or involvement in any organization or entity with any financial interest or non-financial interest in the subject matter or materials discussed in this manuscript.
